# Dynamic Granulocyte Turnover Inferred From cfDNA Methylation Predicts Immunotherapy Benefit in Liver‐Metastatic Small Cell Lung Cancer: A Prospective Biomarker Study

**DOI:** 10.1002/mco2.70925

**Published:** 2026-08-17

**Authors:** Ning Tang, Xiaoling Shang, Yulan Sun, Yingqian Zhang, Haiyan Zhou, Shucheng Ye, Xiaoyong Tang, Jun Guo, Xiqin Zhang, Xuan Gao, Zhehai Wang, Haiyong Wang

**Affiliations:** ^1^ Department of Internal Medicine‐Oncology Shandong Cancer Hospital and Institute Shandong First Medical University and Shandong Academy of Medical Sciences Jinan China; ^2^ Geneplus‐Beijing Institute Beijing China; ^3^ Department of Oncology Affiliated Hospital of Jining Medical University Jining Shandong China

**Keywords:** cfDNA, granulocyte turnover, immune checkpoint inhibitors, liver metastasis, SCLC

## Abstract

Liver metastases (LM) in small cell lung cancer (SCLC) are associated with a limited response to immune checkpoint inhibitors (ICIs). In this trial, we evaluated the efficacy of first‐line envafolimab plus chemotherapy in SCLC patients with LM and explored potential predictive biomarkers. Patients received envafolimab in combination with chemotherapy for four cycles, followed by envafolimab monotherapy until disease progression or unacceptable toxicity. The primary endpoint was progression‐free survival (PFS). Secondary endpoints included safety, objective response, overall survival, and identification of predictive biomarkers. Whole‐exome sequencing (WES), transcriptome sequencing, DNA methylation sequencing, circulating tumor DNA (ctDNA) analysis, and cell‐free DNA (cfDNA) methylation sequencing were performed on tumor tissues and peripheral blood samples. The objective response rate (ORR) was 75.9%, the disease control rate (DCR) was 100%, and the median PFS was 4.3 months. Granulocyte turnover and TNFRSF9 expression emerged as key indicators across transcriptomic and epigenomic analyses, and were associated with prognosis and liver dysfunction. cfDNA‐based assessment of granulocyte turnover reflected both liver damage and tumor dynamics, suggesting its potential for real‐time monitoring. These findings suggest that granulocyte activity may contribute to an immunosuppressive tumor microenvironment and serve as a predictive marker for treatment stratification in SCLC with LM.

## Graphical abstract

This prospective study evaluated envafolimab plus chemotherapy in 29 SCLC patients with liver metastases, integrating multi‐omics analyses (WES, RNA‐seq, DNA methylation, ctDNA, and cfDNA methylation). The research framework encompassed granulocyte turnover dynamics, prognostic risk modeling, molecular mechanism exploration across risk strata, and longitudinal liquid biopsy monitoring.

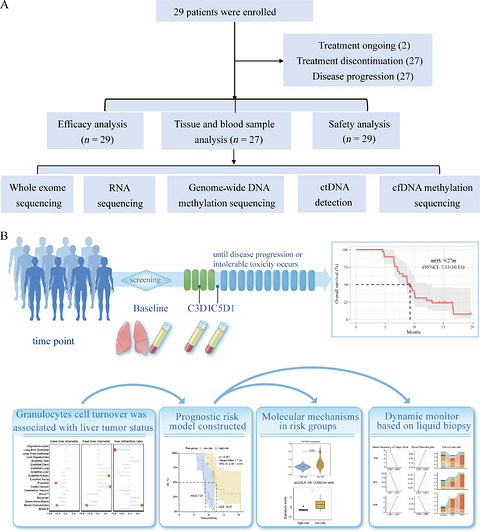

## 1 Introduction

Small cell lung cancer (SCLC), characterized by rapid growth and early metastasis, has a high mortality rate. The prognosis for SCLC patients is dismal, with a 5‐year survival rate of less than 5%. Upon initial diagnosis, nearly two‐thirds of SCLC patients have distant metastases [1]. The liver is one of the most common sites for SCLC metastasis. Approximately 17% of SCLC patients present with liver metastases (LM) upon diagnosis. For extensive‐stage SCLC (ES‐SCLC), the ratio of LM could be up to more than 30% [2]. Unfortunately, SCLC patients with LM is associated with a bleak prognosis [2, 3]. A large cohort study revealed that lung cancer patients with LM had the worst prognosis among the advanced lung cancer cohort, with a mere median overall survival (OS) of 3 months [4]. In another retrospective study including 200 SCLC patients, it is found that the mortality rate is as high as 2.5‐fold in those harboring LM than those without [5].

Although SCLC demonstrated favorable response to platinum‐based chemotherapy initially, with a median OS (mOS) of 10 months, chemoresistance would occur ultimately which ends up in tumor recurrence [6]. With the advent of immunotherapy, the mOS has been extended to approximately 15 months for ES‐SCLC patients [7, 8]. However, the outcomes of several clinical trials regarding ES‐SCLC with LM were less than satisfactory [9, 10, 11, 12]. Further meta‐analyses also indicated that patients with LM did not obtain comparable benefits from the combination of immunotherapy with chemotherapy [13, 14]. Therefore, the therapeutic options for SCLC with LM remain a formidable challenge. While there have not been sufficient studies on the therapeutic options among this particular cohort, identification of exploratory synergistic combination therapies remains an essential unmet clinical need for SCLC patients with LM.

Envafolimab, a subcutaneously administered programmed death‐ligand 1 (PD‐L1) antibody, can reactivate T cells via inhibition of programmed death‐1 (PD‐1)/PD‐L1 pathway, thus recovering antitumor immune response [15]. Several studies have shown that it can improve survival in patients with a variety of advanced tumors with tolerable toxicity, and represents a significant landmark in cancer treatment as it revolutionizes the modes of immunotherapy [16, 17, 18]. One study revealed that envafolimab is effective in Japanese patients with advanced solid tumors in a Phase I study, in which the population was at clinical Stage IV [19]. And it also demonstrates promising efficacy and manageable toxicity as second‐line therapy for metastatic NSCLC [20]. These findings offer us clues that it might exert function in metastatic diseases. Moreover, envafolimab was reported to be effective for chronic hepatitis B in a Phase II clinical trial (NCT04465890), suggesting its immunomodulatory role in the liver [21]. Given these results, prospective studies are needed to verify the role of envafolimab against LM in patients with SCLC. However, currently the efficacy and safety of envafolimab with etoposide and carboplatin/cisplatin in SCLC patients with LM are still elusive.

Tissue turnover has been becoming an up‐to‐date biomarker in disease detection [22, 23]. With the publication and progressive refinement of a comprehensive human cell‑type methylation atlas [24, 25], methylation patterns in cell‑free DNA (cfDNA) have demonstrated convenience and substantial potential in detecting cell death of specific tissues, thereby enabling noninvasive monitoring of various pathological conditions. Peretz et al. reported that DNA methylation‐based liquid biopsies detected vascular endothelial cells related cfDNA biomarkers, informing vascular dynamics in health and disease [26]. McNamara et al. found that cfDNA methylation markers showed promise in monitoring tissue‑specific cellular damage resulting from treatment [27], and similar approaches have been successfully applied to track transplant rejection [28, 29]. Importantly, beyond tissue parenchymal cells, cfDNA methylation can also quantify the turnover of immune cell populations, particularly granulocytes, which represent a key component of the innate immune system. Given the well‑established association between peripheral blood neutrophil‑to‑lymphocyte ratio (NLR) and clinical outcomes in cancer patients receiving immune checkpoint inhibitors (ICIs), we hypothesized that granulocyte turnover inferred from cfDNA methylation might serve as a noninvasive indicator of host immune status and potentially predict immunotherapy benefit in SCLC patients with LM.

In this Phase II trial, we investigated the efficacy and safety of envafolimab plus etoposide and carboplatin/cisplatin as first‑line treatment for ES‑SCLC patients with LM. More importantly, through integrated multi‑omics profiling including whole‑exome sequencing (WES), transcriptome sequencing, genome‑wide DNA methylation, ctDNA analysis, and cfDNA methylation‑based organ‑specific cell turnover assessment, we explored whether baseline granulocyte turnover and its dynamic changes during treatment could predict clinical outcomes. Our findings suggest that granulocyte activity may contribute to an immunosuppressive tumor microenvironment and serve as a potential predictive marker for treatment stratification in this high‑risk patient population.

## 2 Results

### 2.1 Patient Characteristics

**FIGURE 1:**
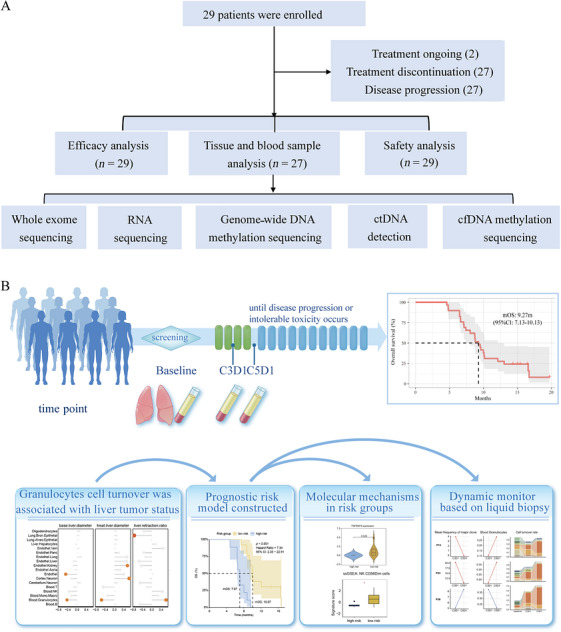
Study design and analytical approach. (A) Patient enrollment and sample analysis: 29 participants were enrolled. During the study, 2 patients remained on treatment while 27 discontinued due to disease progression. Efficacy and safety analyses included all 29 patients, with tissue/blood samples from 27 patients undergoing molecular profiling (whole‐exome sequencing, RNA sequencing, genome‐wide DNA methylation sequencing, ctDNA detection, and cfDNA methylation sequencing). (B) Analytical framework: the study incorporated (i) assessment of granulocyte turnover dynamics relative to tumor status, (ii) development of prognostic risk models, (iii) investigation of molecular mechanisms across risk strata, and (iv) longitudinal monitoring via liquid biopsy approaches.

Between December 2022 and February 2024, a total of 29 ES‐SCLC patients with LM met the eligibility criteria and were enrolled. During the treatment period, 2 patients (6.9%) remained on therapy, while 27 (93.1%) discontinued due to disease progression (Figure 1A). All 29 patients were included in the efficacy and safety analyses. Comprehensive molecular profiling was performed using tissue and blood samples, including WES, RNA sequencing, genome‐wide DNA methylation profiling, ctDNA analysis, and cfDNA methylation‐based organ‐specific cell turnover assessment. These multi‐omics analyses provided an integrated evaluation of treatment efficacy, safety, and underlying molecular mechanisms (Figure 1B).

Building upon the overall cohort description, the baseline clinical features of the 29 enrolled patients are summarized as follows. The median age was 67 years, with 51.7% (15/29) aged ≥ 65 and 48.3% (14/29) aged < 65. The majority of patients were male (79.3%, 23/29), while females accounted for 20.7% (6/29). All patients had a good performance status with an ECOG PS of 0–1 (100.0%). At baseline, 41.4% (12/29) of patients presented with fewer than five LM, and 58.6% (17/29) had five or more. The most common sites of extrahepatic metastases included bone (37.9%, 11/29), adrenal glands (24.1%, 7/29), brain (13.8%, 4/29), and lung (10.3%, 3/29). A comprehensive summary of baseline demographics and disease characteristics is presented in Table 1.

**TABLE 1:** Baseline characteristics of extensive‐stage small cell lung cancer with liver metastases.

Characteristics	Total (*n* = 29)
Age	
≥ 65	15 (51.7%)
< 65	14 (48.3%)
Sex	
Male	23 (79.3%)
Female	6 (20.7%)
ECOG	
0–1	29 (100%)
2	0 (0)
Number of liver metastases	
< 5	12 (41.4%)
≥ 5	17 (58.6%)
Other metastases	
Bone	11 (37.9%)
Brain	4 (13.8%)
Adrenal	7 (24.1%)
Lung	3 (10.3%)

### 2.2 Clinical Efficacy

**FIGURE 2:**
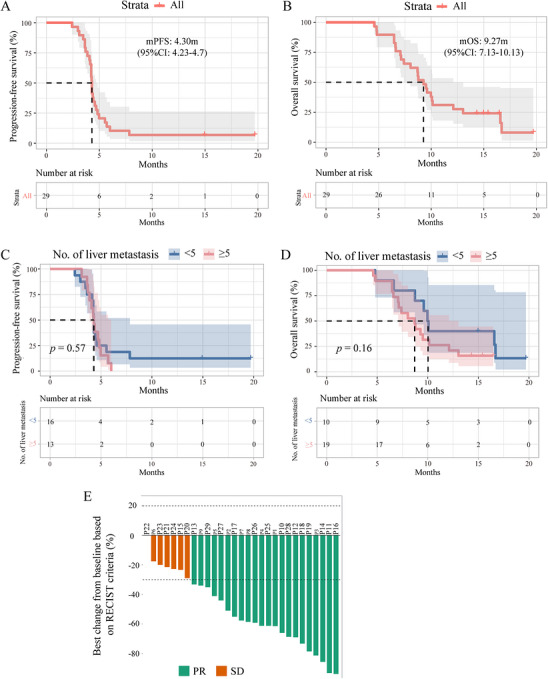
Efficacy outcomes of the study cohort. (A) Progression‐free survival (PFS) for all patients (*n* = 29) receiving envafolimab plus chemotherapy. (B) Overall survival (OS) for all patients (*n* = 29) receiving envafolimab plus chemotherapy. (C) PFS stratified by number of liver metastases (< 5 vs. ≥ 5). (D) OS stratified by number of liver metastases (< 5 vs. ≥ 5). (E) Best tumor response from baseline according to RECIST criteria.

The objective response rate (ORR) was 75.9%, with 22 patients achieving a partial response (PR) and 7 patients demonstrating stable disease (SD), resulting in a disease control rate (DCR) of 100% (Table 2). This indicates encouraging efficacy of the combination therapy in halting disease progression across the cohort. The primary endpoint, median PFS (mPFS) was 4.30 months (95% confidence interval [CI]: 4.23–4.7) (Figure 2A). And the mOS was 9.27 months (95% CI: 7.13–10.13), suggesting a possible long‐term survival benefit (Figure 2B). Subgroup analysis based on the number of LM showed that patients with < 5 LM had an mPFS of 4.283, while those with ≥ 5 LM had an mPFS of 4.30 months (*p* = 0.1188, hazard ratio [HR] = 1.68, 95% CI: 0.72–3.91) (Figure 2C). The mOS of patients with < 5 LM was 9.785, while those with ≥ 5 LM had an mOS of 8.03 months (*p* = 0.1188, HR = 1.68, 95% CI: 0.72–3.91) (Figure 2D). Tumor regression (Figure 2E) visually demonstrated the reduction in tumor size across the patient population, further supporting the efficacy of the treatment regimen.

**TABLE 2:** Efficacy evaluation according to Response Evaluation Criteria in Solid Tumors (RECIST) 1.1.

	Total (*n* = 29)
ORR	22 (75.86%)
DCR	29 (100%)
CR	0 (0)
PR	22 (75.86%)
SD	7 (24.14%)

Abbreviations: ORR, objective response rate; DCR, disease control rate; CR, complete response; PR, partial response; SD, stable disease.

### 2.3 Safety

Treatment‐related adverse events (TRAEs) were reported in all 29 patients (100%). The most common TRAEs were hematologic toxicities, including anemia (93.1%), neutropenia (82.8%), leukopenia (69.0%), and lymphopenia (69.0%). Gastrointestinal reactions were also common, occurring in 44.8% of patients. Most TRAEs were Grade 1 or 2 in severity, with Grade 3 or higher events being less frequent. Grade 3 or higher TRAEs included neutropenia (20.7%), lymphopenia (13.8%), anemia (6.9%), thrombopenia (6.9%), and leukopenia (3.4%). No Grade 4 or 5 TRAEs were reported. The safety data are summarized in Table 3.

**TABLE 3:** Treatment‐related adverse events.

	Total (N/%)	Grade 1/2 (N/%)	Grade ≥ 3 (N/%)
Anemia	27 (93.1)	25 (86.2)	2 (6.9)
Neutropenia	24 (82.8)	18 (62.1)	6 (20.7)
Leukopenia	20 (69.0)	19 (65.5)	1 (3.4)
Lymphopenia	20 (69.0)	16 (55.2)	4 (13.8)
Gastrointestinal reaction	13 (44.8)	13 (44.8)	0
Thrombopenia	10 (34.5)	8 (27.6)	2 (6.9)
Abnormal liver function	6 (20.7)	6 (20.7)	0
Fever	2 (6.9)	2 (6.9)	0
Fatigue	2 (6.9)	2 (6.9)	0
Hypothyroidism	1 (3.4)	1 (3.4)	0

### 2.4 Association Between Cell Turnover and Tumor Burden in SCLC Patients With LM

**FIGURE 3:**
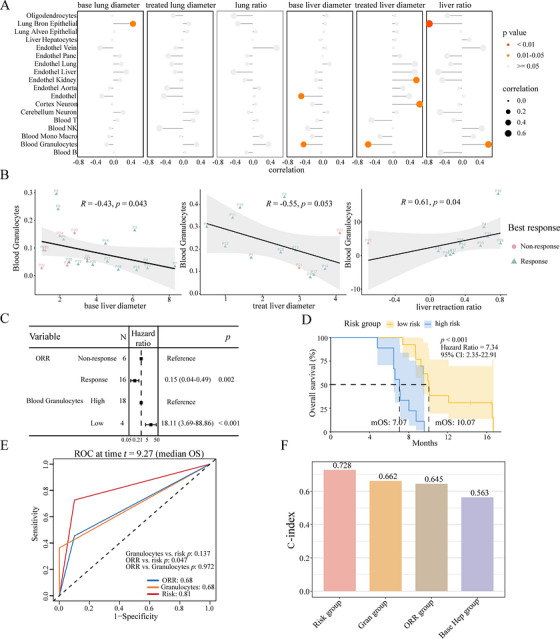
Blood granulocyte turnover correlates with tumor burden and prognosis in SCLC patients with liver metastases (LM). (A) Correlation matrix showing the relationship between blood cell turnover and tumor burden in SCLC patients with LM (*n* = 22). Circle size and line length both indicate correlation strength; orange circles denote significant correlations (p < 0.05) and gray circles denote non‐significant ones. (B) Scatter plot illustrating the correlation between blood granulocyte turnover and corresponding tumor status in the liver. Points are color‐ and shape‐coded to indicate patients’ response status. (C) Forest plot of multivariate Cox proportional hazards model evaluating the association of overall response rate (ORR) and granulocyte turnover with overall survival (OS) based on 22 patients with available baseline cfDNA data (16 responders and 6 non‐responders). (D) Kaplan–Meier survival curves stratified by high‐risk and low‐risk groups derived from the Cox model. (E) Comparison of predictive performance for OS among the risk group, ORR group, and granulocyte turnover group, assessed by area under the curve (AUC) values. (F) Bar plots comparing the C‐index values among the risk group, ORR group, granulocyte group, and base hep group (defined by baseline liver tumor number and size).

Previous studies have demonstrated that SCLC patients with LM often experience rapid disease progression and poor clinical outcomes following treatment [20, 30]. Consistent with these findings, our cohort exhibited a similarly aggressive disease course. To further investigate the underlying biological mechanisms in this subset of SCLC patients, we conducted an integrative multi‐omics analysis. Cell turnover reflects the extent of tissue damage resulting either from tumor infiltration or from host antitumor responses [31, 32]. This dynamic process can be quantitatively inferred through organ‐specific cfDNA methylation signals, referred to as organ‐derived cell fraction (organCF) values. We examined the correlation between organCF levels and tumor burden, measured by lesion diameter, to explore the association between cellular injury and tumor size in both pulmonary and hepatic lesions across different time points. The results revealed that among all the correlation results between organCF and tumor size in lung or liver lesions at different time dimension, only the organCF results of granulocyte kept consistently moderate correlation with tumor in liver (Figure 3A and Figure S1A–C). To be specific, organCF value of granulocyte was negatively associated with liver tumor diameter at baseline and post‐treatment time node respectively (baseline: *r* = −0.43, *p* = 0.043; post‐treatment: *r* = −0.55, *p* = 0.053). And the increased organCF value of granulocyte was positively associated with the retraction of liver tumor diameter (*r* = 0.61, *p* = 0.04) (Figure 3B). These findings provide novel insights into the role of innate immune dynamics in shaping treatment response in SCLC with LM. Increased granulocyte turnover may serve as a surrogate marker of effective tumor regression in the liver.

### 2.5 Prognostic Value of Granulocyte Turnover and Construction of a Risk Stratification Model

Given the strong association between granulocyte turnover and liver tumor dynamics, we next explored its prognostic potential in SCLC patients with LM. To this end, we performed univariate Cox regression analyses incorporating granulocyte organCF values, ORR, and other clinical parameters to assess their impact on OS (Figure S2A). Both ORR and granulocyte turnover emerged as significant predictors of OS. Patients who failed to achieve an objective response exhibited a markedly shorter survival (HR = 3.94, *p* = 0.007; Figure S2B). The optimal cutoff for granulocyte organCF was determined using the surv_cutpoint function based on OS, yielding a value of 0.02696, which was rounded to 0.03 for simplicity and interpretability; patients with values above this threshold showed significantly prolonged OS compared to those below (HR = 9.00, *p* < 0.001; Figure S2C). More importantly, when the analysis was restricted to responders, baseline granulocyte turnover retained its prognostic value. Stratification by the same cutoff yielded a notable difference in survival outcomes (HR = 25.71, *p* < 0.001; Figure S2D), suggesting that granulocyte turnover may further delineate subsets of responders who derive durable benefit from treatment. We next assessed whether these two variables contributed prognostic value independently and multivariate Cox regression analysis confirmed their independent predictive significance (Figure 3C).

Building upon these findings, we integrated granulocyte turnover and ORR into a composite risk model aimed at improving survival prediction in this patient population. Patients with tumor retraction ratio greater than 0.3 and granulocyte organCF value bigger than 0.03 were regarded as low‐risk patients, otherwise were labeled as high risk. For tumor retraction ratio, the clinically established RECIST threshold of 30% tumor reduction (defining the boundary between treatment response and non‐response) was applied. As shown in Figure 3D, low‐risk patients showed longer OS, with a statistically significant difference, with an mOS of 10.07 months. Furthermore, the predictive performance of the composite risk model outperformed either ORR or granulocyte turnover alone. Receiver operating characteristic (ROC) analysis (Figure 3E) and concordance index (C‐index) (Figure 3F) showed that the composite risk model provided potential prognostic discrimination compared to individual biomarkers in this exploratory analysis; however, these findings require prospective validation in independent cohorts.

### 2.6 Distinct Genomic and Epigenomic Profiles Define Risk Groups in SCLC Patients With LM

**FIGURE 4:**
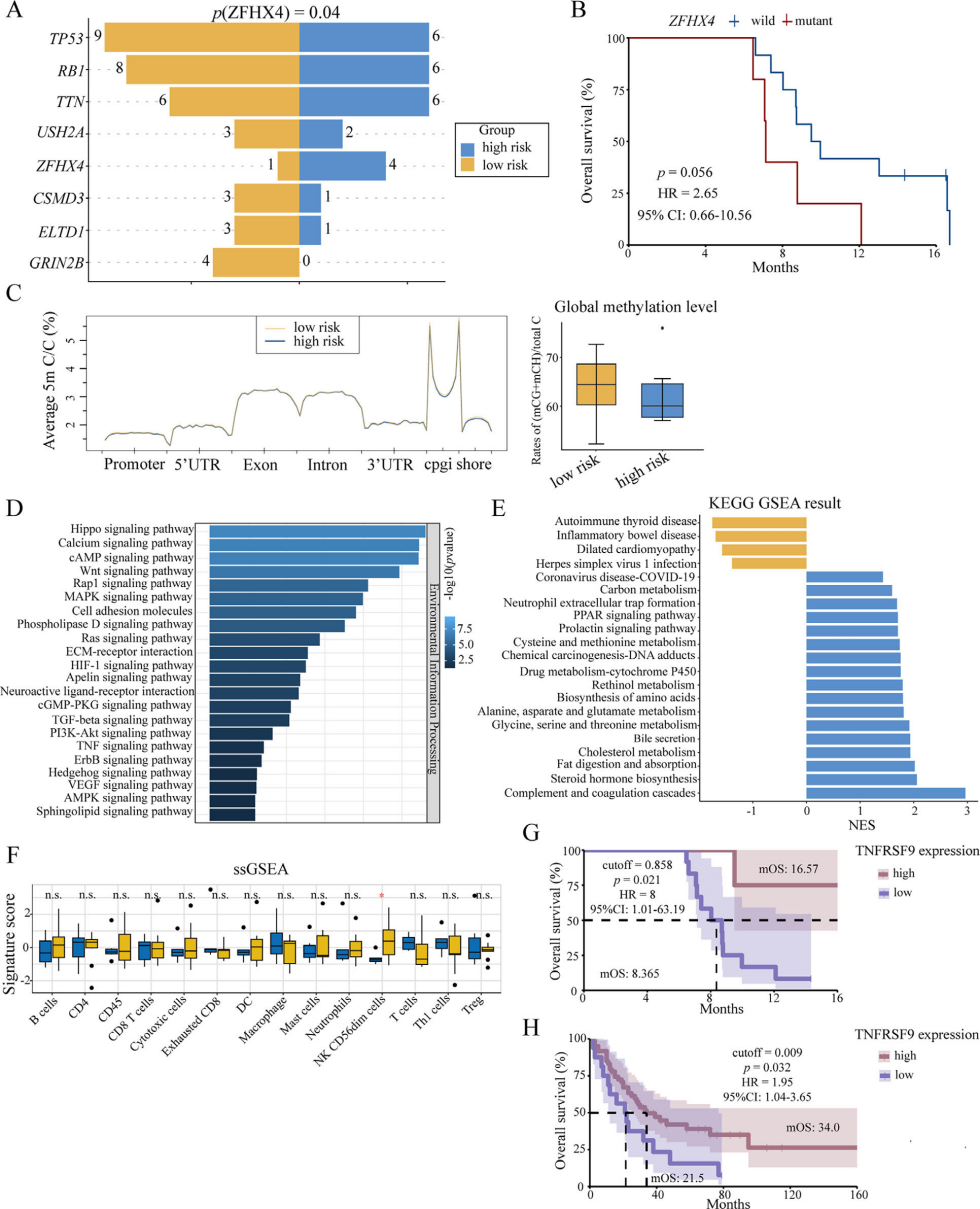
Molecular and immune landscape underlying risk stratification in prognosis in SCLC patients with liver metastases. (A) Mutation frequencies of the top eight annotated genes in high‐risk (*n* = 6) and low‐risk (*n* = 10) patient groups. Numbers within each cell indicate the mutation frequency of each gene within the respective risk group. (B) Kaplan–Meier survival curves comparing patients with wild‐type versus mutant ZFHX4 status (*n* = 16). (C) Comparison of DNA methylation levels between high‐ and low‐risk groups. Left: Mean 5mC/C methylation levels across different genic regions. Right: Boxplots showing global methylation differences between the two risk groups. (D) KEGG pathway enrichment analysis of differentially methylated region (DMR)‐associated genes between high‐ and low‐risk groups. (E) Gene Set Enrichment Analysis (GSEA) of KEGG pathways based on differentially expressed genes (DEGs) between high‐ and low‐risk groups. (F) Immune cell cluster differences between high‐ and low‐risk patients, based on deconvolution of immune infiltration patterns. (G) Kaplan–Meier survival analysis based on high versus low TNFRSF9 expression in the study cohort (*n* = 16). (H) Validation of TNFRSF9‐based stratification in an external cohort (*n* = 120) using Kaplan–Meier survival analysis.

To elucidate the molecular underpinnings of the composite risk model, we compared the primary lung tumors of high‐ and low‐risk patients using WES, transcriptomic profiling, and genome‐wide DNA methylation analysis. Consistent with known molecular features of SCLC, TP53, RB1, and TTN were the most frequently mutated genes in our cohort, occurring in 100%, 89%, and 78% of patients, respectively (Figure S3A). These mutation frequencies were comparable to those observed in our previously published cohort of 178 Chinese SCLC patients, in which TP53, TTN, and RB1 mutations were identified in 87%, 72%, and 62% of patients, respectively [33]. Furthermore, analysis of the cBioPortal Small Cell Lung Cancer cohort [34] revealed the mutation frequencies of these genes were also comparable to those in our cohort (Figure S3B,C). Mutational signature analysis revealed predominant signatures associated with defective DNA mismatch repair and tobacco carcinogen exposure (Figure S3D). Notably, the mutation frequency of ZFHX4 was significantly higher in high‐risk patients compared to low‐risk patients (*p* = 0.04; Figure 4A). ZFHX4 mutations have previously been implicated in predicting response to ICIs and prognosis in NSCLC and melanoma [35]; however, their role in SCLC remains unexplored. In our exploratory analysis, ZFHX4 mutation was associated with worse survival outcomes in ICI‐treated patients with LM (HR = 2.56, *p* = 0.056; Figure 4B), suggesting a hypothesis‑generating observation that warrants validation in larger cohorts. We further compared ZFHX4 RNA expression levels between mutant and wild‐type groups in 18 patients with available transcriptomic data. No significant difference in expression was observed, although the median FPKM value was relatively higher in the wild‐type group (Figure S3E). Consistent with this finding, analysis of the cBioPortal Small Cell Lung Cancer cohort also revealed no significant difference in ZFHX4 mRNA expression between mutant and wild‐type groups (Figure S3F). In the present cohort, the overall mutation frequency of ZFHX4 was 39%, consistent with frequencies reported in other SCLC cohorts [33, 34]. We further assessed the association between ZFHX4 mutation status and ORR but observed no significant difference between mutant and wild‐type groups (Figure S3G), suggesting that ZFHX4 mutation may be more closely associated with long‐term prognosis rather than short‐term treatment response in this patient population.

To further contextualize the transcriptomic features of our cohort, we classified patients according to the established ANPI subtypes based on RNA sequencing data. Among these, approximately 79% were classified as Type A, 5% each as Type N and Type P, and 10% as mixed subtypes (Figure S4A). Notably, only three patients with Type A subtype showed non‐response to treatment (Figure S4B). Patients with Type A subtype exhibited relatively worse OS (Figure S4C). However, given the extremely limited sample size‐with only one case each for Type N and Type P—these findings are strictly exploratory and hypothesis‑generating, and no definitive conclusions can be drawn from this subtype analysis.

We then explored genome‐wide DNA methylation patterns and transcriptional profiles between risk groups to uncover additional molecular features linked to immunotherapy response. Global methylation analysis revealed a trend toward global hypomethylation in high‐risk tumors, particularly in enhancer and open chromatin regions (Figure 4C), potentially reflecting epigenetic dysregulation associated with immune evasion. And differentially methylated regions (DMRs) (Figure S5A) were enriched in many canonical cancer‐related pathways, such as Wnt signaling, MAPK signaling, TGF‐beta signaling, PI3K‐Akt signaling and so on (Figure 4D).

Next, we performed differential analyses and enrichment analyses based on expression matrix to explore the mechanism of gene regulation and immune filtration in the TME. Interestingly, we noticed that in the KEGG Gene Set Enrichment Analysis (GSEA) results (Figure 4E), the neutrophil extracellular trap (NET) formation pathway and the PPAR signaling pathway were upregulated in high‐risk group. PPARα plays an important role in liver function, and study has reported that PPARα agonist can improved cholestatic liver injury by attenuating inflammatory response and reducing neutrophil infiltration [36]. Immune cell deconvolution via single‐sample GSEA (ssGSEA) further revealed a distinct infiltration pattern: the low‐risk group exhibited higher abundance of CD56 dim NK cells, a cytotoxic subset linked to favorable ICI responses (Figure 4F). Among the differentially expressed immune‑related genes identified between risk groups, TNFRSF9 was selected for further investigation based on its established role as a co‑stimulatory immune checkpoint (4‑1BB/CD137), its ongoing clinical evaluation as a therapeutic target, and its previously reported association with ICI responsiveness. Notably, risk group comparison of immune gene signatures revealed that TNFRSF9 expression was markedly downregulated in high‑risk patients, with expression undetectable in this group (Figure S5B). As a known costimulatory receptor under active investigation in cancer immunotherapy [37], this finding may represent a novel biomarker of immune competence in the SCLC LM context.

To further evaluate the prognostic relevance of TNFRSF9 expression in SCLC, we analyzed OS based on TNFRSF9 expression levels in both our cohort and an independent external SCLC cohort comprising 120 patients [34]. In both cohorts, low TNFRSF9 expression was significantly associated with worse OS (Figure 4G for our cohort; Figure 4H for the external validation cohort). Notably, the optimal cutoffs in both analyses were close to zero, reinforcing that the complete lack of TNFRSF9 expression observed in high‐risk patients was not due to technical artifact but represents a biologically relevant and reproducible phenomenon. To further validate the prognostic significance of TNFRSF9 at the protein level, we performed immunohistochemistry (IHC) staining in formalin‐fixed paraffin‐embedded (FFPE) tumor tissues from 19 patients with ES‐SCLC who received first‑line immunotherapy. Based on IHC scores, patients were divided into high (*n* = 8) and low (*n* = 11) TNFRSF9 expression groups (Figure S5C). Consistent with the transcriptomic findings, high TNFRSF9 protein expression was associated with significantly longer OS (*p* = 0.012) and PFS (*p* = 0.019) (Figure S5D,E), providing complementary evidence that TNFRSF9 may serve as a prognostic biomarker in this patient population.

### 2.7 Granulocyte Turnover Integrates Liver Injury and Immune Dysfunction in SCLC Patients With LM

To further explore the potential interactions among molecular markers, immune pathways, and liver‐related phenotypes in patients with differential risk profiles, we performed correlation analyses across several key variables. These included pathway activity scores (NETs formation and PPAR signaling from ssGSEA), immune cell infiltration (ssGSEA score for NK CD56 dim cells), stimulatory immune checkpoint expression (TNFRSF9 mRNA level), clinical biochemical indicators (NEU, ALT, AST, AST/ALT), and cfDNA‐derived cell turnover signals (blood granulocytes, liver epithelial cells, and hepatocytes). The results, summarized in Figure S6, revealed a complex but coherent network of associations. Most patients, particularly those in the high‐risk group, exhibited elevated ALT, AST, and AST/ALT ratios, consistent with liver dysfunction. Turnover of liver hepatocytes showed strong correlations with these biochemical indices, suggesting that hepatocellular death is a primary contributor to hepatic injury. Furthermore, liver dysfunction indicators were significantly associated with dysregulated PPAR signaling, reduced NK CD56 dim cell infiltration, and suppressed TNFRSF9 expression, pointing to a close interplay between liver damage and an immunosuppressive TME.

Importantly, granulocyte turnover emerged as a central node in this network, showing significant correlations with liver function markers, PPAR signaling, NK CD56 dim cell infiltration, and TNFRSF9 expression. These findings imply that increased granulocyte activity may act as a key intermediary in the pathological axis of liver tumor progression, hepatic damage, and immune dysfunction. This multi‐parametric correlation landscape suggests that granulocyte turnover plays a pivotal role in shaping the immunobiology and treatment responsiveness of SCLC patients with LM.

### 2.8 Dynamic Monitoring of Tumor Burden via ctDNA and organCF

**FIGURE 5:**
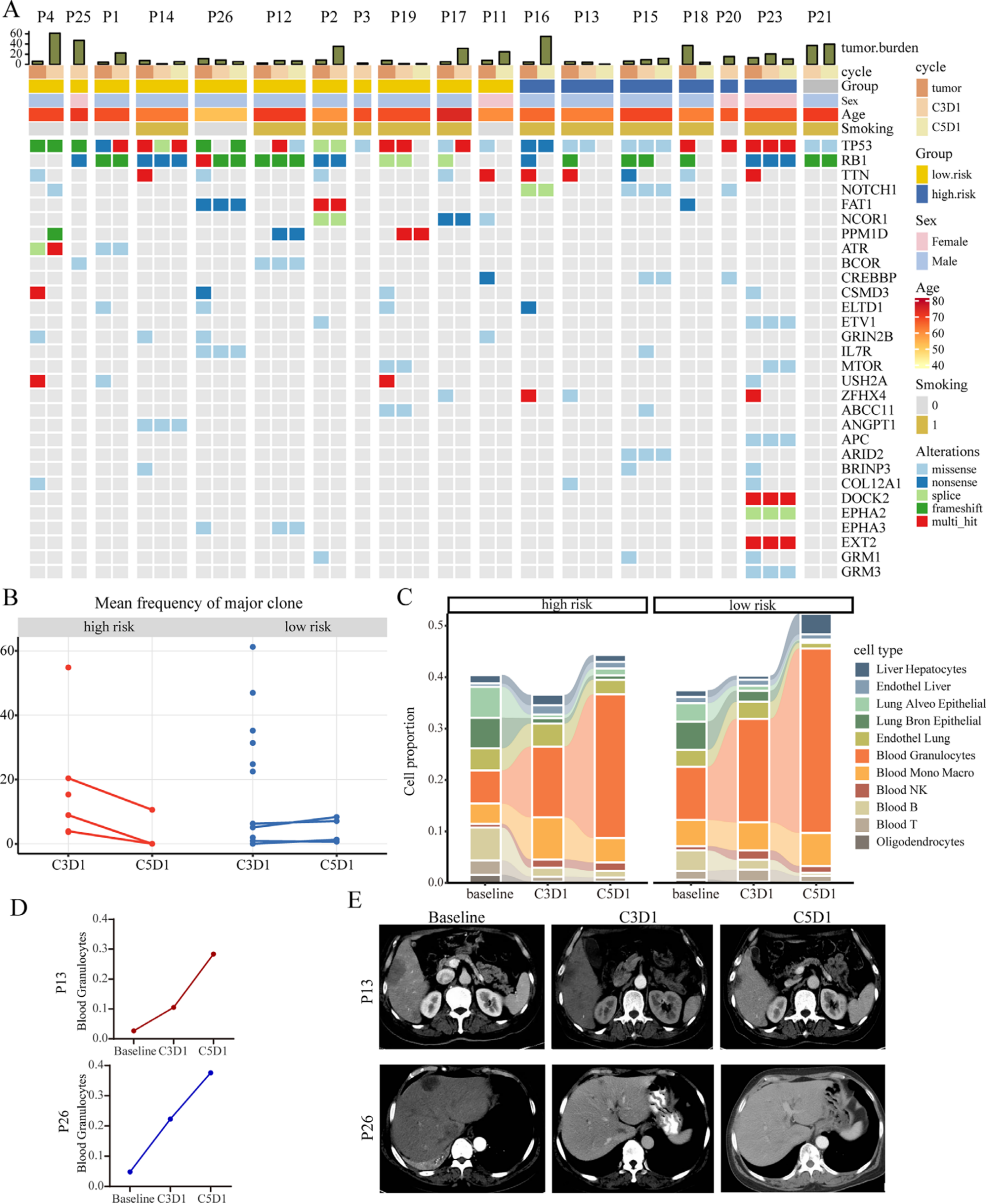
Dynamic clonal and cellular turnover profiles associated with treatment response prognosis in SCLC patients with liver metastases. (A) Integrated landscape of clinical characteristics and functional mutations across samples collected at baseline (tumor tissue), C3D1 (blood), and C5D1 (blood). (B) Longitudinal changes in the mean frequency of the dominant tumor clone from C3D1 to C5D1 in high‐ and low‐risk patients. Each line represents an individual patient. (C) Sankey plots illustrating the dynamic changes in mean organ‐specific cell‐free DNA contribution (organCF) for selected cell types from baseline to post‐treatment in high‐ and low‐risk groups. (D) Longitudinal trajectories of organCF values in circulating granulocytes from baseline to C3D1 and C5D1 in two representative patients (P13 and P26). (E) The computed tomographic imaging of liver metastases lesion of P13 and P26 who received envafolimab plus chemotherapy.

To track tumor burden and tissue‐specific cell turnover after treatment, we analyzed cfDNA through ctDNA profiling for tumor alterations and organCF scoring for methylation‐based turnover assessment. Consistent with tumor tissue analysis, the most frequently detected mutations in ctDNA were found in TP53 and RB1. Longitudinal analysis of maximum allele frequency (MAF) from paired plasma samples after Cycle 3 Day 1 (C3D1) and Cycle 5 Day 1 (C5D1) revealed that patients with shorter PFS tended to exhibit increased MAF over time (Figure S7A). We further mapped functional mutations captured at baseline in tumor tissue and tracked their persistence in ctDNA at C3D1 and C5D1 (Figure 5A), showing that most mutations, once identified in the tumor, remained detectable in circulation throughout treatment.

In terms of treatment monitoring, cfDNA concentration demonstrated a general downward trend during therapy in both high‐ and low‐risk groups (Figure S7B). To explore clonal dynamics, we employed PyClone to infer subclonal architecture from ctDNA, and the paired clone traits change of each patient can be found in Figure S7C. The mean allele frequency (AF) of the dominant clone (defined in Section 4 Methods) evolved differently across risk groups. Specifically, in high‐risk patients, the dominant clone showed a reduction in AF from C3D1 to C5D1, whereas in low‐risk patients, the dominant clone's AF increased over the same interval (Figure 5B). Quantification of this change using the C5D1/C3D1 ratio of mean AF revealed a borderline significant difference between risk groups (*p* = 0.057; Figure S7D), suggesting distinct clonal evolution patterns in response to therapy.

Next, we observed that the organCF of lung, liver, and immune related cells were changed in line with the treatment's development. As we can see from Figure 5C, died cells from lung epithelial were remarkably decreasing after treatment, turnover of immune cells (such as granulocytes, monocytes, and macrophage) has increased, while the death rate of B cells, T cells, and oligodendrocytes shown descending trends. Together, these results of organ parenchymal cells and immune cells, we inferred that our medicine has an effect on protecting lung and influencing the immune infiltration, especially the granulocytes.

To illustrate the relationship between granulocyte turnover and therapeutic efficacy, we longitudinally tracked organCF values and tumor burden in two representative cases: Pt. 26 (low‐risk group) and Pt. 13 (high‐risk group). Granulocyte organCF values increased over time in both patients; however, the magnitude of elevation was notably greater in Pt. 26, suggesting a more robust granulocyte turnover response (Figure 5D). Consistent with this observation, serial imaging revealed that Pt. 26 experienced marked shrinkage of LM lesion at both the early treatment phase (C3D1) and after four cycles (C5D1), compared to baseline. In contrast, Pt. 13 exhibited limited tumor regression in hepatic lesions over the same period (Figure 5E). These findings further support the notion that enhanced granulocyte turnover is closely associated with superior tumor regression in SCLC patients with LM receiving envafolimab plus chemotherapy.

## 3 Discussion

To the best of our knowledge, this was the first prospective Phase II study to evaluate the efficacy of envafolimab plus chemotherapy in a particular cohort of ES‐SCLC with LM. Our study demonstrated its favorable response with a manageable safety profile.

Several studies of first‐line immunotherapy for ES‐SCLC have enrolled patients with LM. ASTRUM‐005 study revealed that serplulimab plus chemotherapy improved OS compared to mere chemotherapy for SCLC with LM [10]. However, in IMpower 133, atezolizumab did not bring significant OS benefit for the unique cohort of LM [11]. KEYNOTE‐604 and CAPSTONE‐1 study also demonstrated that no OS benefit was brought by the addition of pembrolizumab or adebrelimab to chemotherapy [12, 38]. In contrast, our regimen achieved a rather favorable clinical efficacy and safety profile of the combination of envafolimab plus chemotherapy. Notably, the ORR was 75.86% (95% CI, 72.8%–78.7%), and the DCR was 100% (95% CI, 94.1%–100%). These suggested that our combination treatment may be of therapeutic value for SCLC patients with LM.

Metastasis serves as one of the major causes of cancer associated mortality. Liver is one of the most frequent sites where SCLC metastasize. The liver is generally viewed as an immune‐tolerant organ owing to its T‐cell anergy and immunosuppressive signals. These properties contribute to an immune desert, resulting in negative response to immunotherapy and shorter survival in patients with LM, which have been observed in both preclinical models and patients [39, 40]. Another study proposed that the TME in LM of lung cancer exhibits low immune activation, which leads to a poor organ‐specific tumor response to immunotherapy [41].

A series of meta‐analyses have been performed to evaluate the role of ICI in treating SCLC patients with LM. A study led by Lee et al. demonstrated that the progression of LM may have influenced ICI resistance, contributing to dismal survival [42]. In another study led by Chen et al., the addition of ICIs to chemotherapy significantly improved OS in ES‐SCLC patients with LM compared with chemotherapy alone [14]. And the study by Xia et al. revealed that LM may undermine the OS benefit in patients with SCLC [13]. In a series of landmark high‐quality clinical trials, as exemplified by CASPIAN, ASTRUM‐005, RATIONALE‐312, and ETER701 studies, results also vary with regard to the role of ICI in patients with LM [9, 10, 43, 44]. Therefore, understanding the role of ICI in immune regulation is indispensable for effective therapeutics in SCLC patients with LM.

Our study explores on the clinical translation potential of biomarkers to predict risk of SCLC patients with LM. First, we reported the robust association between granulocytes and liver tumor. We then found that granulocytes turnover at baseline can predict dismal survival of patients, not only in all patients we recruited but also the patients classified as responder for treatment. We further constructed a risk model based on granulocytes turnover and ORR, and investigated the molecular characteristics in primary lesion to dig out the underlying mechanisms in high‐risk patients. It turns out that patients identified as high risk by our biomarkers have shown specific gene expression (like stimulatory immune checkpoint TNFRSF9) and related pathways pattern (like NETs and PPAR), as well as different immune cell infiltration in primary lesion compared with low‐risk patients. This approach could identify SCLC patients with higher risk for LM and might precede radiological recurrence. Finally, we explored the potential of ctDNA and cfDNA methylation pattern in monitoring patients’ outcome. This study represents a novel application of cfDNA methylation‐based cell turnover analysis for predicting and monitoring clinical outcomes. These findings were further supported by complementary multi‐omics data, revealing exploratory insights and suggesting promising biomarkers that warrant further clinical investigation.

Our results suggest that granulocyte dynamics are intricately linked to both tumor progression and treatment response. Granulocyte death in circulation was associated with liver tumor burden and impaired liver function, potentially through modulation of TNFRSF9 expression, disruption of the PPAR signaling pathway, and altered infiltration of NK CD56 dim cells. Notably, baseline granulocyte turnover showed prognostic relevance, indicating its potential utility in predicting clinical outcomes. In parallel, treatment appeared to exert a dual effect‐preserving lung tissue integrity while mobilizing granulocytes back into the TME, potentially enhancing local immune activity. In recent years, granulocytes, particularly neutrophils, have been recognized as key mediators of TME [45]. The important role of granulocytes in the pathogenesis of cancers has been reported in hepatocellular carcinoma (HCC) [46, 47], colorectal cancer (CRC) LM [48], breast cancer lung metastasis [49], and some other types [50]. Considering our results that patients with lower granulocytes death at baseline had bigger liver tumor size and shorter OS, and that baseline granulocyte turnover correlated with the sum of LM diameters but not with the number of liver lesions, we inferred that these undead granulocytes may act as drivers of tumor progression, which corresponds to reports that some tumor‐associated neutrophil populations were associated with an unfavorable prognosis in patients with liver cancer [47, 51]. Xiao et al. discovered the mechanism of cancer metastasis as tumor‐secreted protease cathepsin C promotes breast‐to‐lung metastasis by regulating recruitment of neutrophils and NETs [49]. Another study reported roles of IL‐17 and Wnt signaling pathway in granulocytes in CRC LM [48]. Those results may offer explanations to our findings, as we also reported the key role of granulocytes in our cohort. Yet more exploration is needed for underlying mechanisms in SCLC with LM.

Our findings carry potential implications for clinical practice. Dynamic changes in immune cell populations, particularly granulocyte turnover, emerged as potential indicators of metastatic risk, offering a minimally invasive means to stratify patients. For those already presenting with LM, our data suggest that targeting the immunosuppressive TME could enhance the efficacy of immunotherapy. Such approaches could potentially benefit from integration into precision medicine frameworks. Nevertheless, larger, prospective cohorts are needed to validate the utility of these multi‐omic biomarkers in predicting response to ICIs and guiding clinical decision‐making.

This study has several limitations that should be acknowledged. One major limitation is the inability to obtain LM tissue at baseline, which constrained our ability to further investigate the molecular alterations and potential biomarkers specific to the liver TME. In addition, although granulocyte turnover demonstrated prognostic relevance—particularly in distinguishing high‐ and low‐risk responders and thereby informing precision stratification—the limited sample size reduced the statistical power of subgroup analyses and restricts the generalizability of these findings. The generalizability of the WES findings is similarly constrained, especially after stratification into risk groups. The modest sample size inherently limited the power to detect robust mutational associations. Thus, observed differences in mutation frequencies, such as for ZFHX4, should be interpreted as hypothesis‐generating rather than definitive, and validation in larger independent cohorts is required. Finally, while our data suggest the promise of using dynamic changes in cell turnover and ctDNA metrics to monitor treatment response and predict long‐term outcomes, the current sampling schedule and the number of longitudinal samples were insufficient for comprehensive temporal tracking. Future studies with expanded cohorts and more frequent sampling time points are warranted to validate and deepen these observations.

Despite these limitations, our study provides preliminary great clinical value for SCLC patients with LM. It provides preliminary data on the efficacy and safety of envafolimab plus etoposide and carboplatin/cisplatin as first‐line treatment for ES‐SCLC patients with LM. Besides, this integrated multi‐omics dataset holds promise for identifying potential biomarkers predictive of response to envafolimab in SCLC patients with LM, while offering deeper insights into the complex tumor microenvironment characteristic of this subgroup.

## 4 Methods

### 4.1 Patients

Patients with histologically or cytologically confirmed ES‐SCLC with LM according to the Veterans Administration Lung Study Group (VALG) staging system were enrolled. Additional main inclusion criteria included: age ≥ 18 years and ≤ 75 years; presence of at least one measurable lesion by imaging according to the RECIST version 1.1; no prior systemic therapy or ICI treatment for ES‐SCLC; and no symptoms of central nervous system involvement or no worsening of symptoms within the past 2 weeks. The critical exclusion criteria included: Previously received any of the following therapies: anti‐PD‐1, anti‐PD‐L1, or anti‐PD‐L2 agents, or drugs targeting other stimulatory or co‐inhibitory T‐cell receptors; received systemic therapy with traditional Chinese medicine or immunomodulatory drugs; and presence of clinically uncontrollable pleural effusion or ascites.

### 4.2 Trial Design and Treatments

This study was a prospective, single‐arm Phase II clinical trial. Patients with ES‐SCLC with LM who provided informed consent and met the eligibility criteria would receive first‐line treatment with envafolimab in combination with etoposide and cisplatin/carboplatin. After four cycles, patients continued with envafolimab monotherapy until disease progression or intolerable toxicity. Tumor response was assessed using RECIST v1.1 criteria, with imaging evaluations every 6 weeks during the first year and every 9 weeks thereafter. Safety was assessed using NCI‐CTCAE v5.0, with evaluations every 3 weeks until disease progression, withdrawal of informed consent, loss to follow‐up, or death.

Enrolled patients were given envafolimab (400 mg, subcutaneous injection, D1, Q3W, continued until disease progression or intolerable toxicity) and chemotherapy (platinum agent, selected by the investigator, administered every 3 weeks for a total of four cycles. Cisplatin: 25 mg/m^2^ per day on Days 1–3. Carboplatin: AUC 5, D1, with a maximum dose not exceeding 750 mg. Etoposide: 80–100 mg/m^2^, D1–3, Q3W for a total of 4 cycles). Baseline assessment was conducted within 28 days before the first administration of the study drug. Starting from the first administration of the study drug, tumor imaging evaluations were conducted every 6 weeks (±7 days). After 1 year, imaging evaluations were conducted every 9 weeks (±7 days). Safety follow‐up was conducted 30 ± 7 days after the last dose of the study drug or before the initiation of a new antitumor treatment, whichever occurred first. Survival follow‐up was conducted every 90 days (±7 days) after the safety follow‐up visit.

### 4.3 Efficacy Evaluation

The primary analysis of efficacy was based on the Full Analysis Set (FAS), which was defined as all patients who had received at least one dose of the study drug and from whom at least one efficacy assessment after treatment was available. For missing efficacy data, the last observation carried forward (LOCF) method was used for imputation, and the common analytical method was used for the description of quantitative or count data. For ORR and DCR, in addition to providing the point estimate of the difference between the two groups, the 95% CI estimate of the difference was also provided.

### 4.4 Safety Evaluation

Safety was analyzed based on the Safety Set (SS), which was defined as subjects who had received the study drug and had a safety evaluation after medication. Safety analysis mainly consisted of descriptive statistical summaries. Statistical summaries were made for adverse events (AEs), treatment‐emergent AEs (TEAEs), SAEs, as well as laboratory data, vital signs, and other data.

### 4.5 Sample Preparation

DNA and RNA from pulmonary primary lesion were extracted using the AllPrep DNA/RNA FFPE Kit. Peripheral blood samples were separated to obtain plasma and peripheral blood lymphocytes (PBLs), then cfDNA was extracted from plasma using MagMAX cfDNA Isolation Kit, and germline genomic DNA (gDNA) was obtained from PBLs using CWE9600 Blood DNA Kit.

### 4.6 WES and Data Analysis

DNA sheared into fragments with a peak of 250 bp was used for library construction, and DNA sequencing was conducted on the DNBSEQ‐T7RS platform (BGI, Shenzhen, China) with 100 bp paired‐end sequencing strategy. Raw sequencing data were mapped to the human reference genome (GRch.37) and sorted using BWA software after removing low‐quality reads by Fastp, then duplications were marked and filtered out by Picard. Single nucleotide variants (SNVs) and insertions or deletions (INDELs) were called by MuTect2. Variants were kept if they met the following criteria: (i) detected in at least five high‐quality reads; (ii) with variant AF > 0.02; and (iii) with AF < 0.01 in databases including ExAC, dbSNP, or 1KG.

### 4.7 RNA‐seq and Gene Expression Data Analysis

mRNA was reversely transcribed to cDNA and amplified by PCR, then the mRNA libraries were paired‐end sequenced on DNBSEQ‐T7RS sequencer (BGI, Shenzhen, China). After trimming adapter and removing low‐quality reads, clean reads were mapped to GRch.37 by STAR. The processed expression matrix and group info were submitted to perform differential analysis using DESeq2, pathway enrichment analysis and GSEA using clusterProfiler, immune cell scores and stromal scores using ESTIMATE R package, immune cell infiltration using different algorithms including ssGSEA, TIMER, CIBORSORT, MCPcounter, EPIC, and xCell. ANPI subtyping was performed according to the classification scheme originally described by George et al. Briefly, RNA‑seq expression data for ASCL1, NEUROD1, POU2F3, and YAP1 were extracted and normalized. Patients were classified into Type A (ASCL1‑dominant), Type N (NEUROD1‑dominant), Type P (POU2F3‑dominant), or mixed subtypes based on the relative expression dominance among these four transcription factors.

### 4.8 Genome‐Wide DNA Methylation Sequencing and Data Processing

The methylation libraries were conducted by oxidizing 5mC to 5caC through TET2 oxidase, then transformed into dihydrouracil under the action of the reductant Pyridine borane. Paired‐end sequencing was performed using DNBSEQ‐T7RS sequencing platform (BGI, Shenzhen, China). Raw sequencing data were filtered and mapped to GRch.37, then methylation sites were called by asTair. The DMRs were analyzed by methylKit R package with default parameters. DMR was identified as regions (1000 bp width interval) with absolute differential methylation levels > 0.1, CpG coverage ≥ 5, CpG number ≥ 10 per region, and distance < 300 bp between adjacent CpGs. Next, DMRs were annotated to functional regions by genomation R package, and pathway enrichment analysis was performed based on DMRs‐related genes.

### 4.9 ctDNA Detection

Libraries were constructed using 170 bp cfDNA and 200∼250 bp gDNA, respectively, then hybridized to a custom‐designed 1021 panel and sequenced on DNBSEQ‐T7RS platform (BGI, Shenzhen, China). After standardized protocol for quality control and filter, true somatic mutations were kept and samples with at least one variant reported were defined as ctDNA‐positive. Note that somatic mutations were remained following below steps: (i) the variants not present in matched gDNA; (ii) the single‐nucleotide polymorphisms at > 1% population AF; (iii) the depth on target without duplicated reads was at least > 200×. ctDNA sequencing was performed using a custom‑designed 1021‑gene panel. The average sequencing depth on target (after duplicate removal) was 2045× (range: 616×–5007×), providing sufficient coverage for reliable variant detection. For PyClone‑based subclonal inference, only somatic mutations with adequate read support and variant AF were retained. The 1021‑gene panel covers a broad range of cancer‑related genes and has been validated in multiple ctDNA‑based clonal evolution studies, ensuring sufficient mutation sites for robust subclonal reconstruction. The dominant clone was defined for each patient as the mutation with the highest VAF in the earliest available ctDNA sample (C3D1, or C5D1 if C3D1 was unavailable), and its VAF was tracked longitudinally to monitor clonal dynamics.

### 4.10 cfDNA Methylation Sequencing and Organ Source Tracing

Blood samples for cfDNA analysis were collected at protocol‐defined time points prior to each treatment cycle (baseline, Cycle 3 Day 1 [C3D1], and Cycle 5 Day 1 [C5D1]), always before the administration of study treatment on the day of collection. For patients who received granulocyte colony‐stimulating factor (G‐CSF) during the treatment period, blood collection was scheduled at least 7 days after the last G‐CSF administration. Candidate DNA methylation sequencing of cfDNA was performed on platform called GM‐seq, the cfDNA was hybridized to a custom‐designed panel which was designed to infer damage of targeted organ combining with prior DNA methylation atlas knowledge. Before library construction, sequences with CpG totally methylated (positive references) and CpG totally unmethylated (negative references) were mixed into the samples as controls. After we obtained the clean methylation data stored in BAM file, we used in‐house developed deconvolution algorithm FragDedup to detect pattern of DNA methylation, and used Maximum Likelihood Estimation (MLE) model to infer quantitate fraction of cell derived from specific organs which named organCF by us, suggesting turnover ratio of specific cell released into blood.

### 4.11 Association Between organCF and Tumor Size

For each blood sample, organCF values for 49 cell types were calculated. A total of 22 baseline samples were obtained (16 from patients with subsequent PR, 6 from those with SD), and 17 post‐treatment samples (10 after C3D1 and 7 after C5D1). For the tumor size results, we collected the maximum diameter of tumor observed in lung and liver, respectively. Considering the sample sparsity in C3D1 and C5D1 time node, we merged organCF and tumor size results of C3D1 and C5D1 for the same patient. As we can see the drug effect almost occurred after two cycle treatment, we only selected C5D1 results in the situation that there had no C3D1 results for the patients. Next, we calculated the Spearman's rank correlation between paired organCF and tumor size of lung and liver in baseline and after‐treatment time node, respectively. We also explored the relationship between tumor retraction ratio and the changes of organCF from baseline to post‐treatment, the equation is stated as follow:

$$\begin{array}{ccc} \text{Tumorsizechange} & = & (\text{Baseline}\text{value}-\text{Post}-\text{treatment}\text{value})/\\ & & \;\;\text{Baseline}\text{value}\times 100. \end{array}$$

organCFchange=Post−treatmentvalue/Baselinevalue.

### 4.12 IHC Staining

FFPE tumor tissue sections from 19 patients with ES‐SCLC who received first‐line immunotherapy were used for TNFRSF9 protein detection. After standard deparaffinization, antigen retrieval, and blocking, sections were incubated with an anti‐TNFRSF9 (abcam) primary antibody, followed by a horseradish peroxidase‐conjugated secondary antibody and diaminobenzidine (DAB) visualization. TNFRSF9 expression was evaluated independently by two experienced pathologists who were blinded to clinical outcomes. Based on staining intensity and the proportion of positive tumor cells, patients were classified into high‐expression (*n* = 8) and low‐expression (*n* = 11) groups.

### 4.13 Statistical Analyses

For categorical variables were frequencies (percentage [%]). PFS and OS were estimated using the Kaplan–Meier method. All tests were two‐sided, with the significance level *α* set at 0.05. The CI, unless otherwise specified, was at the 95% confidence level. Statistical analysis was conducted using SAS version 9.4. Patients who had not experienced disease progression or death at the time of analysis were censored at the time of the last tumor assessment. Group comparisons were conducted using Student's *t*‐test or Mann–Whitney test, depending on the variables and corresponding data distribution. The optimal cutoff of organCF value to group patients was calculated based on Kaplan–Meier curve by surv_cutpoint function from R package “survminer.” Univariate and multivariate Cox regression models were performed to analyze the relationship between variables and PFS/OS. Variables with *p* < 0.05 in univariate Cox regression analysis were candidates for inclusion in the multivariate model. A two‐tailed *p*  <  0.05 was considered statistically significant.

## Author Contributions

H.Y.W. and Z.H.W. contributed to project administration, conceptualization, resource allocation, and funding acquisition. N.T., X.L.S., and Y.L.S. contributed equally to this work. They were responsible for original draft preparation, data analysis, methodology design, and supervision of clinical execution. Y.Q.Z. and X.G. were involved in multi‐omics data analysis, bioinformatics interpretation, and manuscript revision. H.Y.Z., S.C.Y., and X.Y.T. contributed to patient enrollment, clinical data collection, and follow‐up management. J.G. and X.Q.Z. participated in statistical analysis and interpretation of clinical endpoints. All authors have read and approved the final manuscript.

## Funding

This study was supported by the Joint TCM Science & Technology Projects of National Demonstration Zones for Comprehensive TCM Reform (GZY‐KJS‐SD2023‐074), the Shandong Provincial Natural Science Foundation General Project (ZR2023MH065), the Shandong Provincial Traditional Chinese Medicine Key Discipline Construction Project (2022) 4, the Collaborative Academic Innovation Project of Shandong Cancer Hospital, the China Postdoctoral Science Foundation (2026M792314), and Postdoctoral Innovation Program of Shandong Province (SDCX‐ZG‐202601041).

## Ethics Statement

This study was approved by the Ethics Committee of Shandong Cancer Hospital and Institute (Approval No. SDZLEC2022‐241‐01). All patients provided written informed consent prior to enrollment. The study was conducted in accordance with the Declaration of Helsinki, Good Clinical Practice (GCP) guidelines, and applicable local regulations. This trial has been registered on ChiCTR with the identifier ChiCTR2400092388.

## Conflicts of Interest

Yingqian Zhang is an employee of Geneplus‐Beijing Institute, but has no potential relevant financial or non‐financial interests to disclose. The other authors declare no conflicts of interest.

## Supporting information




**Supporting file 1**: mco270925‐sup‐0001‐SuppMat.pdf

## Data Availability

The raw sequencing data have been deposited in the Genome Sequence Archive in National Genomics Data Center, China National Center for Bioinformation / Beijing Institute of Genomics, Chinese Academy of Sciences (GSA‑Human: HRA019967) that are publicly accessible at https://ngdc.cncb.ac.cn/gsa‑human. All data that support the findings of this study are available by contacting the corresponding author at hywang8316@sdfmu.edu.cn.
