# Supplementary material for: Dynamic Granulocyte Turnover Inferred From cfDNA Methylation Predicts Immunotherapy Benefit in Liver‐Metastatic Small Cell Lung Cancer: A Prospective Biomarker Study
Source: MedComm (2020). 2026 Aug 17;7(9):e70925. doi: 10.1002/mco2.70925 (PMC13478802; doi:10.1002/mco2.70925)
Supplement: Supplementary file 1 [file MCO2-7-e70925-s001.pdf]

Supplementary material

Dynamic Granulocyte Turnover Inferred from cfDNA Methylation Predicts Immunotherapy Benefit in Liver-Metastatic Small-Cell Lung Cancer: A Prospective Biomarker Study

Author order: Ning Tang [1#], Xiaoling Shang [1#], Yulan Sun [1#], Yingqian Zhang [2], Haiyan Zhou [1], Shucheng Ye [3], Xiaoyong Tang [1], Jun Guo [1], Xiqin Zhang [1], Xuan Gao [2], Zhehai Wang [1*], Haiyong Wang [1*]

Affiliations:

[1] Department of Internal Medicine-Oncology, Shandong Cancer Hospital and Institute, Shandong First Medical University and Shandong Academy of Medical Sciences, Jinan 250017, China.

[2] Geneplus-Beijing Institute, Beijing, China.

[3] Department of Oncology, the Affiliated Hospital of Jining Medical University, Jining, Shandong, China.

*Corresponding authors:

Zhehai Wang and Haiyong Wang

Department of Internal Medicine-Oncology, Shandong Cancer Hospital and Institute, Shandong First Medical University and Shandong Academy of Medical Sciences, Jinan 250117, China.

Tel: 860531-87984777; Fax: 86531-87984079;

E-mail:

Badgood007@126.com for Zhehai Wang

hywang8316@sdfmu.edu.cn for Haiyong Wang

[#] These authors have contributed equally to this work.

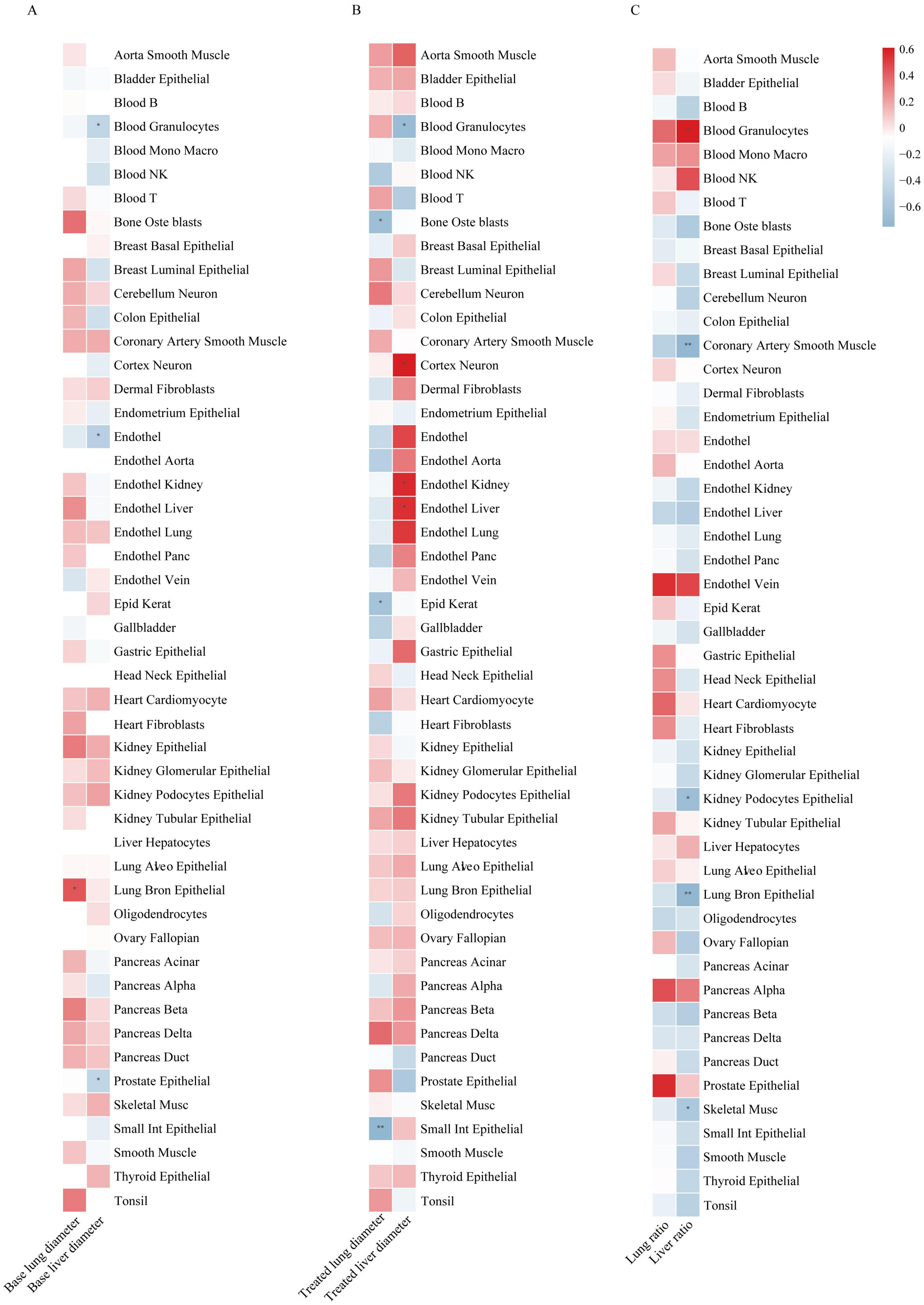

Figure S1 **A** The correlation between 49 types of organCF value and primary lesion diameter, liver metastasis lesion diameter at baseline. **B** The correlation between 49 types of organCF value and primary lesion diameter, liver metastasis lesion diameter after treatment of Envafolimab plus chemotherapy. **C** The relationship between

primary tumor or liver metastasis retraction ratio and the changes of organCF from baseline to post-treatment.

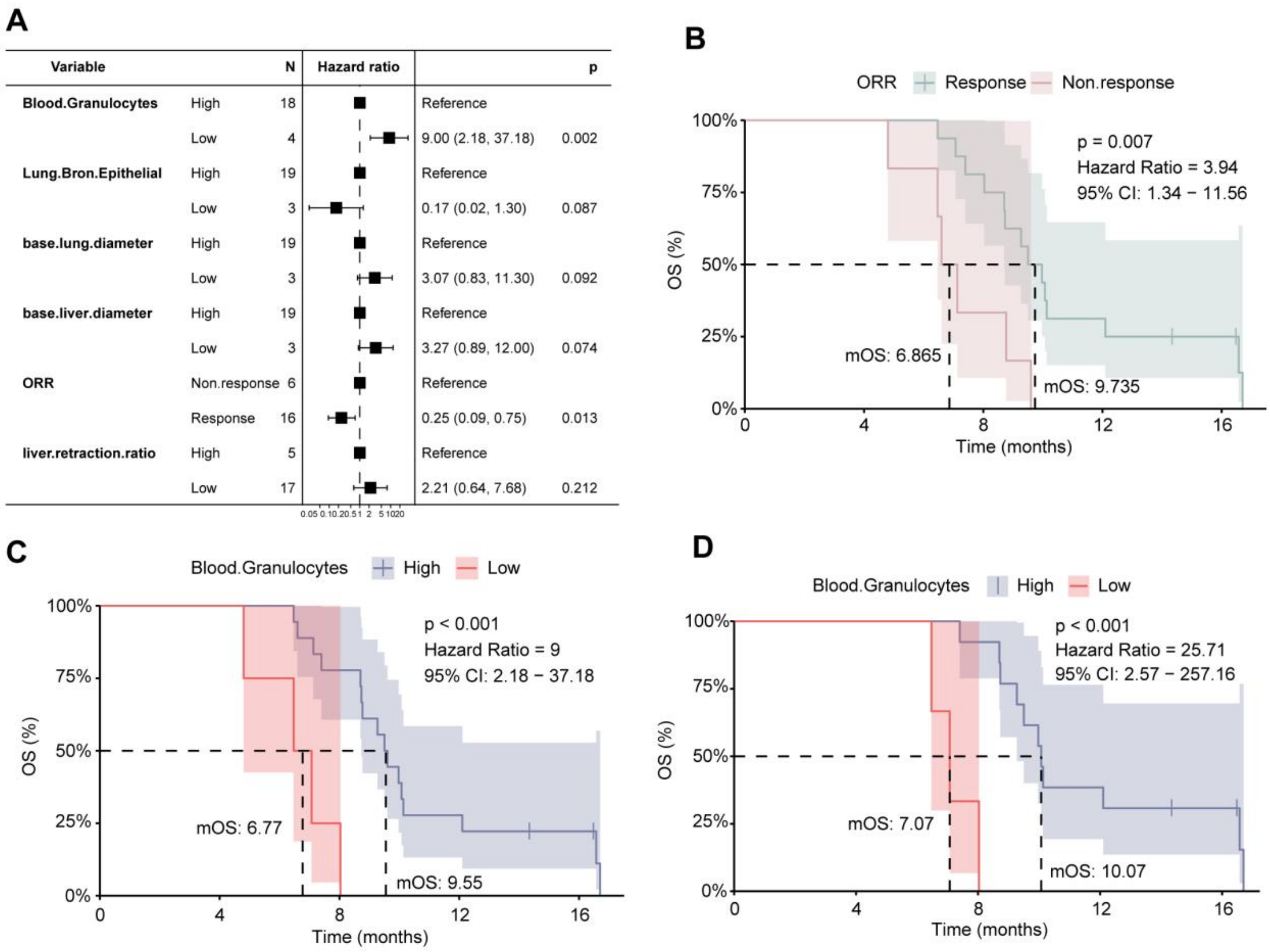

Figure S2 Prognostic Impact of Clinical Response and Granulocyte Dynamics Prognosis in SCLC Patients with Liver Metastases. A Univariate Cox regression analysis of various clinical and translational factors associated with overall survival (OS). **B** Kaplan-Meier survival curves comparing responders and non-responders (n = 22). **C** Kaplan-Meier curves stratified by high versus low blood granulocyte-derived organCF values (n = 22). **D** Kaplan-Meier survival analysis among responders, further stratified by high versus low granulocyte organCF values (n = 16).

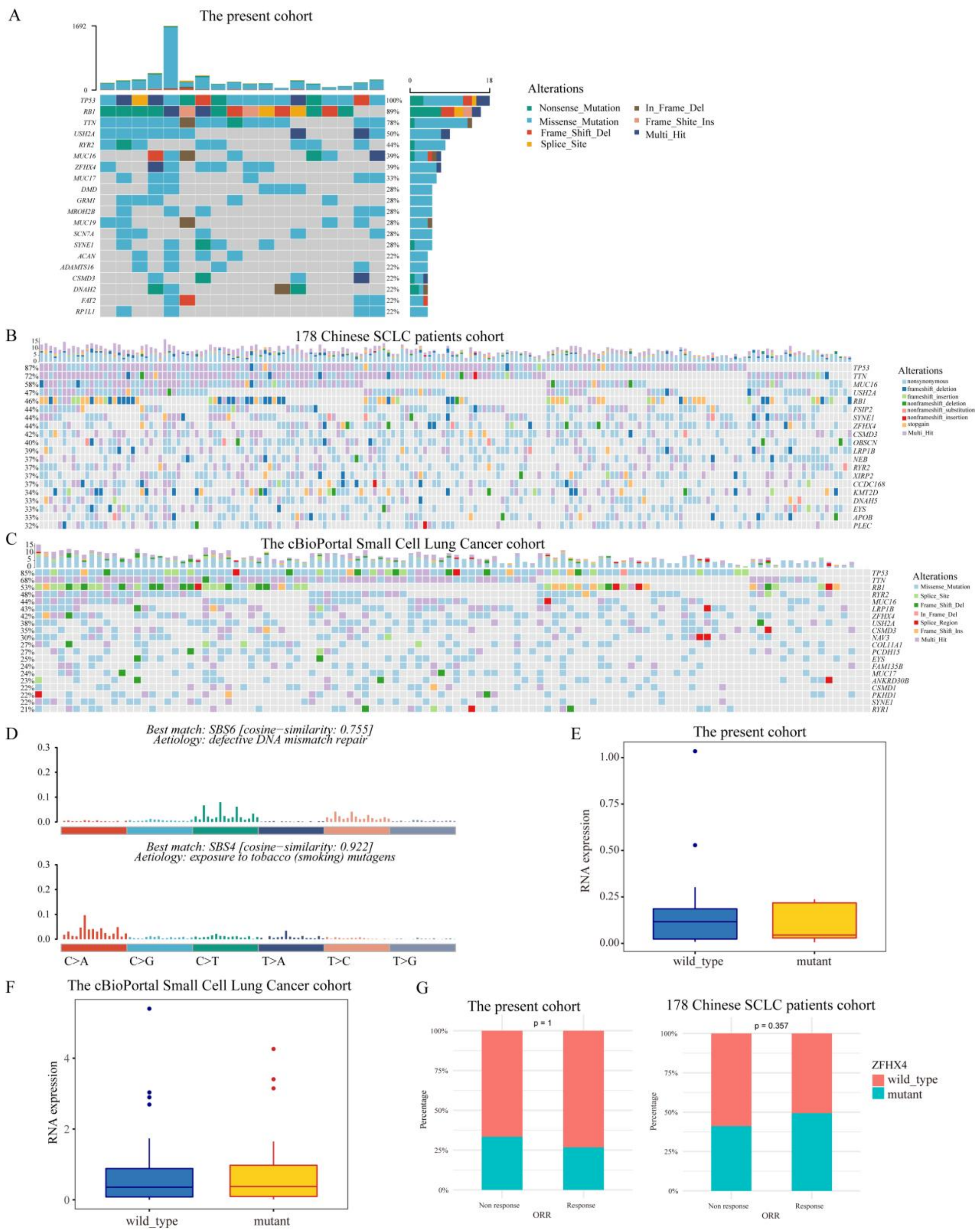

Figure S3 Genomic, Epigenetic, and Immune Profiling of Baseline Lung Tumors Prognosis in SCLC Patients with Liver Metastases. A Landscape of functional somatic mutations detected in baseline lung tumor samples (n = 17). **B** Mutation landscape of an independent Chinese SCLC cohort (n = 178) showing the most frequently mutated genes. **C** Mutation landscape of the cBioPortal Small Cell Lung Cancer cohort showing comparable mutation frequencies. **D** Mutational signature profiles identified in baseline lung tumor samples (n = 17), based on trinucleotide

context analysis. **E** Boxplot comparing ZFHX4 RNA expression levels between ZFHX4 mutant and wild-type groups in the present cohort (n = 18). **F** Boxplot comparing ZFHX4 RNA expression levels between ZFHX4 mutant and wild-type groups in the cBioPortal Small Cell Lung Cancer cohort. **G** Association between ZFHX4 mutation status and ORR in the present cohort and the Chinese SCLC cohort.

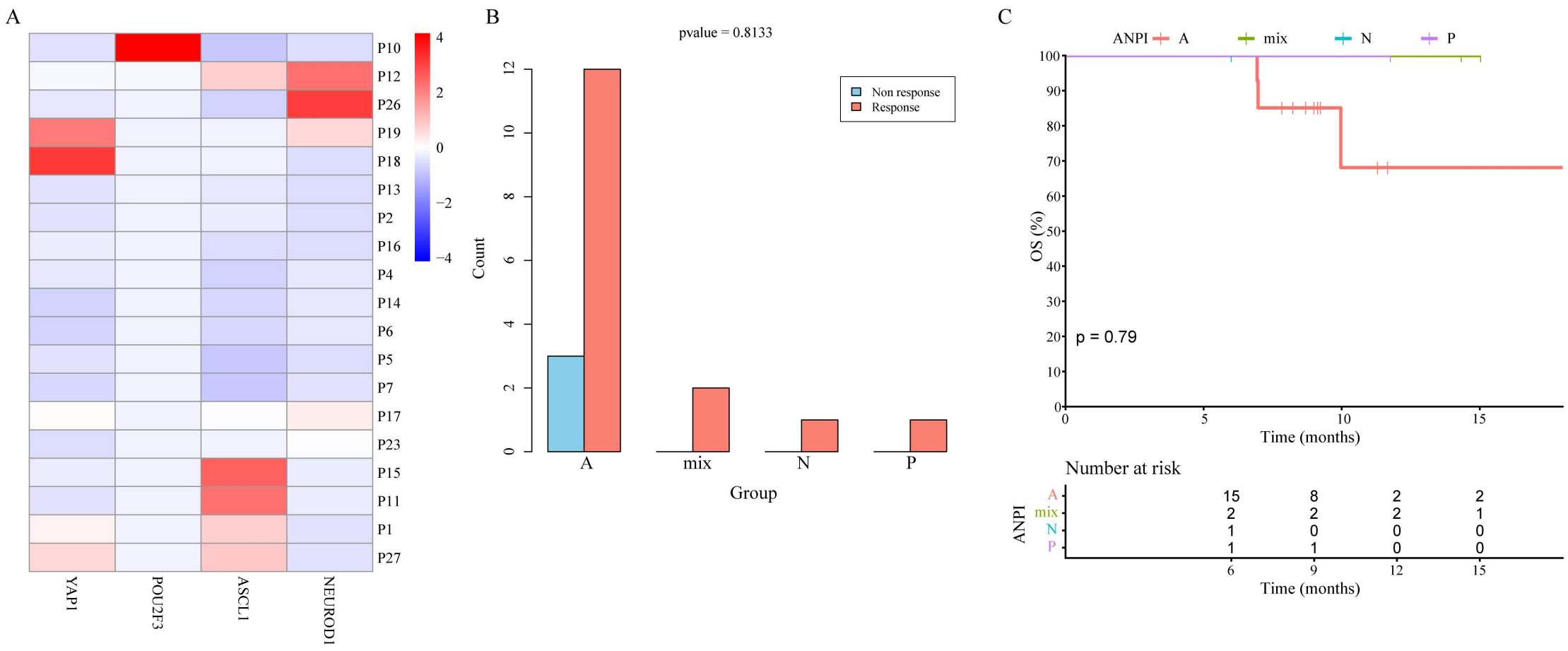

Figure S4 Overview of APNI subtype classification and its association with treatment response and patient survival. A Heatmap showing the expression levels of four subtype-defining transcription factors (ASCL1, NEUROD1, POU2F3, and YAP1) across individual patients. **B** Bar chart displaying the number of responders and non-responders in each subtype. **C** Kaplan-Meier survival curves for each subtype.

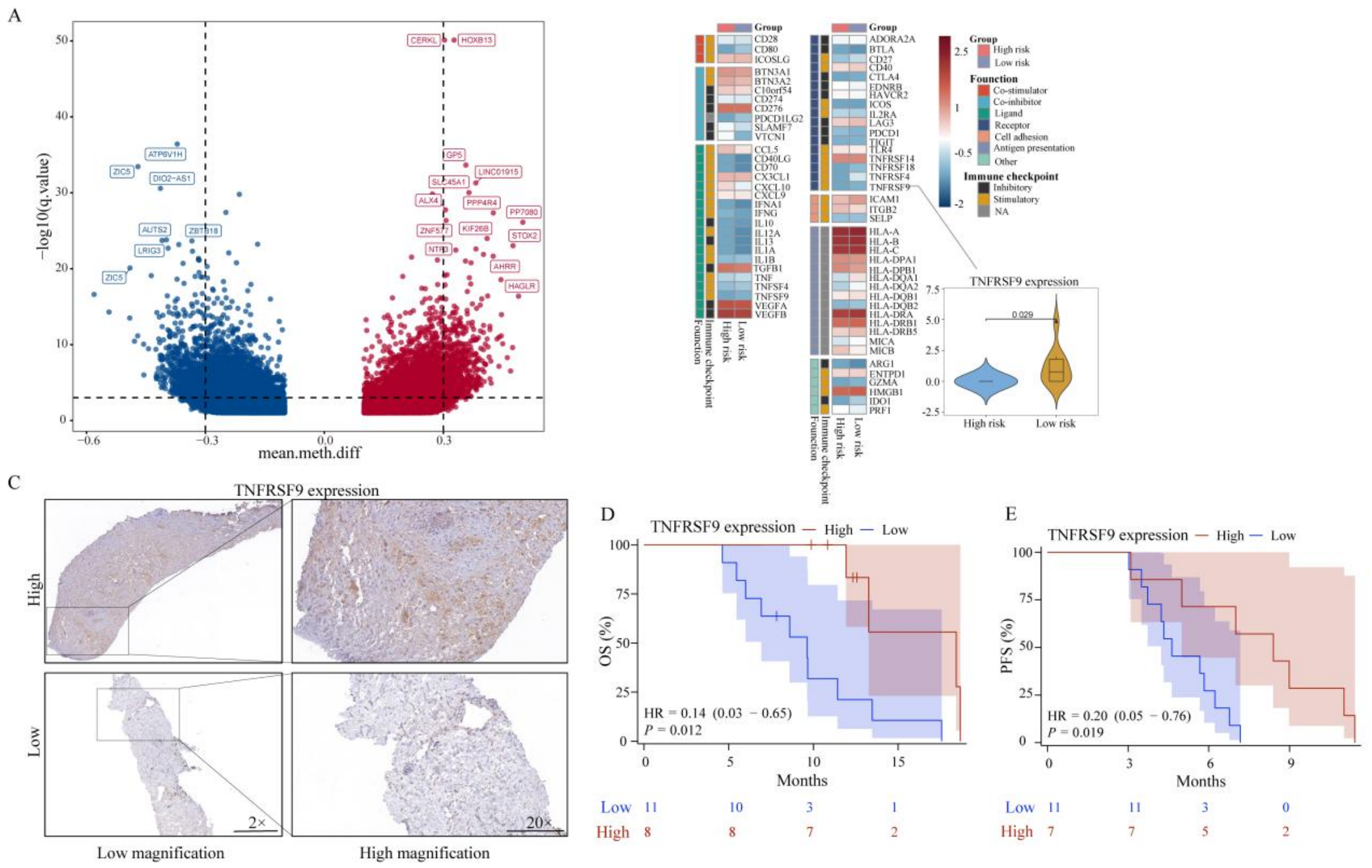

Figure S5 Differential methylation, immune-related gene expression, and TNFRSF9 protein analysis. A Volcano plot of differentially methylated region (DMR)-associated genes, highlighting genes with significant methylation changes. **B** Heatmap of expression levels for 70 selected immune-related genes in high- and low-risk groups. A boxplot comparing TNFRSF9 expression between the two groups is shown on the right. **C** Representative immunohistochemistry images of patients with high and low TNFRSF9 expression. **D** Association between TNFRSF9 protein expression and overall survival (OS). **E** Association between TNFRSF9 protein expression and progression-free survival (PFS).

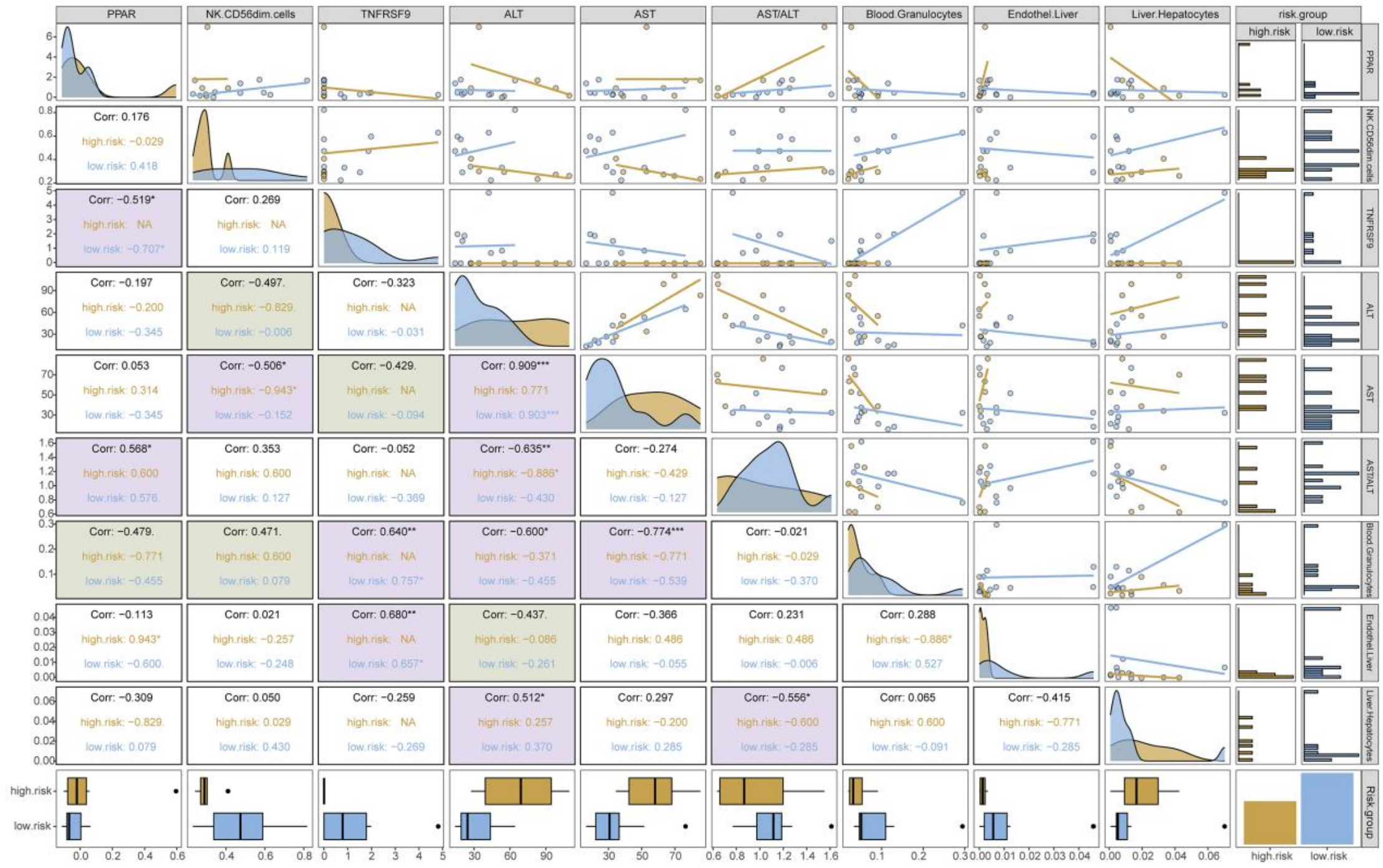

Figure S6 Integrated Correlation Landscape of Biochemical Markers, Pathways, and Cell Turnover Prognosis in SCLC Patients with Liver Metastases. Combined plots displaying the distribution and boxplots of various biochemical indicators, molecular markers, signaling pathways, and cell turnover parameters. Pairwise correlations among these variables are illustrated in the bottom-left correlation matrix. Background shading indicates correlation significance: light purple highlights statistically significant correlations ($P < 0.05$), while light green denotes trends toward significance ($0.05 \leq P < 0.1$).

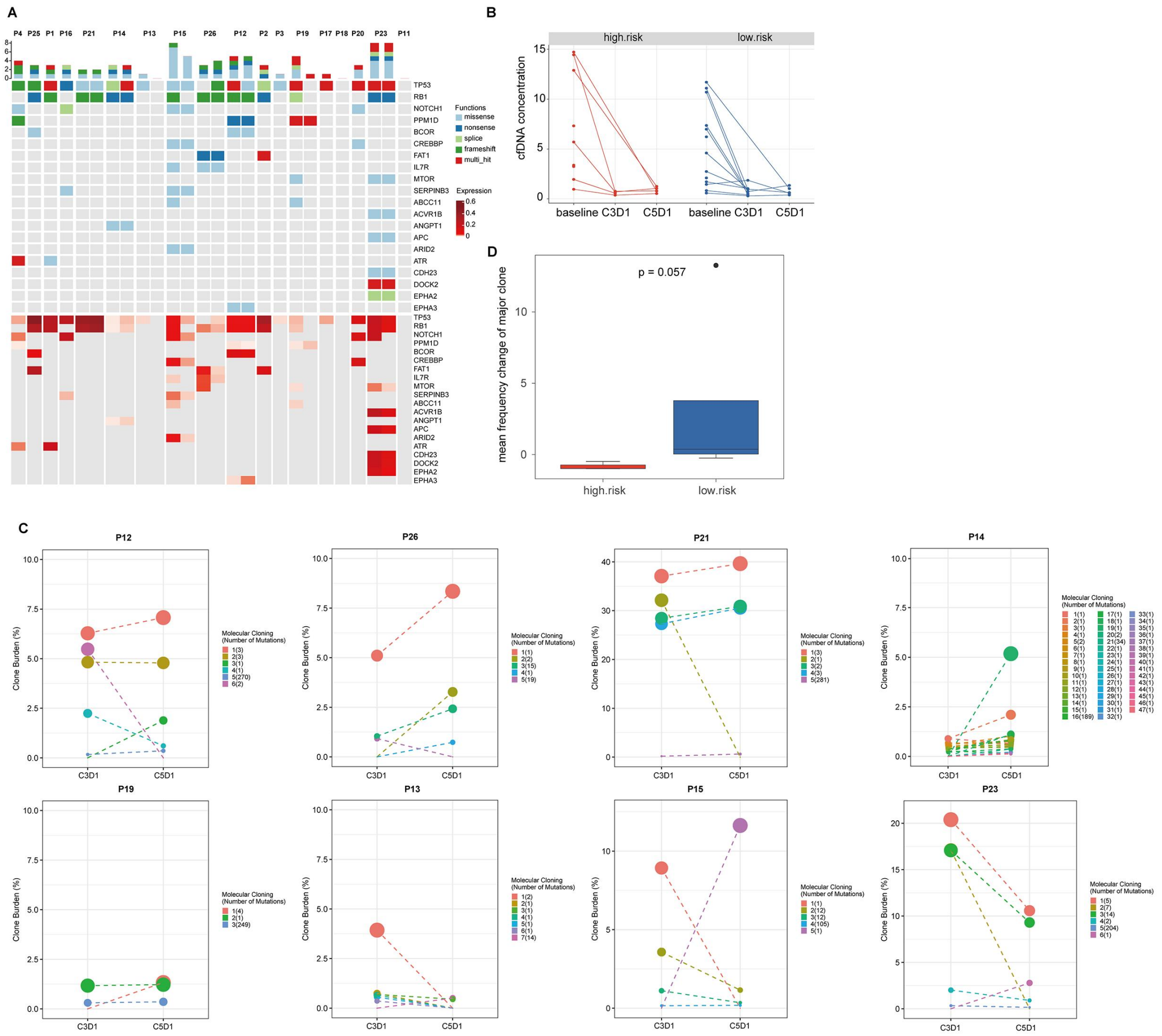

Figure S7 Dynamic ctDNA and Clonal Evolution Profiles during Treatment Prognosis in SCLC Patients with Liver Metastases. A Landscape of functional mutations detected in blood samples at C3D1 and C5D1 timepoints. The heatmap below displays the corresponding mutant allele frequencies (MAFs) for each mutation. **B** Longitudinal changes in cell-free DNA concentration from baseline to post-treatment in high- and low-risk groups. Each line represents an individual patient. **C** Clone burden plots showing the distribution of clonal populations in each patient across timepoints. **D** Boxplot comparison of changes in the mean frequency of the dominant clone between high- and low-risk groups.
